# Use of formative research in developing a knowledge translation approach to rotavirus vaccine introduction in developing countries

**DOI:** 10.1186/1471-2458-7-281

**Published:** 2007-10-05

**Authors:** Evan Simpson, Scott Wittet, Josefina Bonilla, Kateryna Gamazina, Laura Cooley, Jennifer L Winkler

**Affiliations:** 1 PATH, 1455 NW Leary Way, Seattle, WA 98107 US; 2 Federación Red NicaSalud, Managua, Nicaragua; 3 PATH-Ukraine, 2A Bankova Street, 01601, Kyiv, Ukraine

## Abstract

**Background:**

Rotavirus gastroenteritis is the leading cause of diarrheal disease mortality among children under five, resulting in 450,000 to 700,000 deaths each year, and another 2 million hospitalizations, mostly in the developing world. Nearly every child in the world is infected with rotavirus at least once before they are five years old.

Vaccines to prevent rotavirus or minimize its severity are now becoming available, and have already been introduced into the public vaccine programs of several Latin American countries. The World Health Organization (WHO) has made rotavirus vaccine introduction in developing countries a high priority.

The WHOs Guidelines for Vaccine Introduction indicates that a key determinant to achieving vaccine introduction is the public health priority of the disease, suggesting that where the disease is not a priority uptake of the vaccine is unlikely. WHO recommends conducting a qualitative analysis of opinions held by the public health community to determine the perceptions of the disease and the priority given to the vaccine.

**Methods:**

This paper presents the formative research results of a qualitative survey of public health providers in five low- and middle-income countries to determine if and to what degree rotavirus is perceived to be a problem and the priority of a vaccine. Open-ended surveys were carried out through focus group discussions and one-on-one interviews.

**Results:**

Researchers discovered that in all five countries knowledge of rotavirus was extremely low, and as a result was not considered a high priority. However, diarrhea among young children was considered a high priority among public health providers in the three poorest countries with relatively high levels of child mortality: India, Indonesia, and Nicaragua.

**Conclusion:**

In the poorest countries, advocacy and communication efforts to raise awareness about rotavirus sufficient for prioritization and accelerated vaccine introduction might benefit from a knowledge translation approach that delivers information and evidence about rotavirus through the broader context of diarrheal disease control, an existing priority, and including information about other new interventions, specifically low-osmolarity oral rehydration solution and zinc treatment.

## Background

Despite impressive public health gains made in the 1980s and 1990s, severe dehydration caused by diarrheal disease still contributes significantly to childhood morbidity and mortality in the developing world. Today, diarrheal disease is responsible for an estimated 1.6 to 2.4 million deaths annually [1], making it the second leading infectious disease killer of children under five. Among diarrheal diseases, rotavirus gastroenteritis is a leading cause of morbidity and mortality in this population.

Each year, rotavirus is responsible for an estimated 450,000–700,000 deaths among children under five, and another 2 million are hospitalized [2]. Over 80 percent of rotavirus deaths occur in developing countries, where access to emergency treatment such as intravenous rehydration is limited. The primary mode of rotavirus transmission is fecal-oral, and it causes rapid dehydration due to vomiting and diarrhea.

A unique feature of rotavirus is its global scope. Nearly every child in the world, regardless of geography, socio-economic status, or gender will get at least one rotavirus infection before age five [3].

Among diarrheal diseases, rotavirus is an exception to the management rules. Traditional diarrheal disease prevention efforts, such as improving hygiene, sanitation, and access to clean water, are not sufficiently effective in preventing rotavirus, as evidenced by the near universal rate of infection. And, because of the profuse and persistent vomiting that usually accompanies severe cases, oral rehydration therapy is a very difficult treatment to successfully administer. A vaccine is considered to be the most effective prevention method.

The World Health Organization (WHO) has made the development and distribution of rotavirus vaccines in developing countries a high priority [4]. As a result, manufacturers, governments, and global health organizations are working together with the GAVI Alliance to accelerate the availability of safe and efficacious rotavirus vaccines for children in developing countries [5].

In 2006, a three-dose oral rotavirus vaccine developed by Merck & Co. Inc. was approved for use by the U.S. Food and Drug Administration (FDA). In addition, a two-dose oral vaccine developed by GlaxoSmithKline was approved by the European equivalent of the FDA, the European Agency for the Evaluation of Medicinal Products. Both vaccines have demonstrated high levels of safety and efficacy in major clinical trials in the U.S., Latin America, and Europe [6,7], and as a result the WHO's Strategic Advisory Group of Experts (SAGE), has indicated that adoption of rotavirus vaccines in these regions is warranted [8]. In June, 2007, the GAVI Alliance announced it would subsidize the cost of rotavirus vaccines to GAVI eligible countries (those with a gross national income of less than US$1,000 per capita) in Latin America and Europe, making these vaccines available to these countries at US$0.30 per course [9].

As of June, 2007, four non-GAVI eligible countries in Latin America (Brazil, Panama, Venezuela, and El Salvador) have introduced rotavirus vaccine into their Expanded Program on Immunization (EPI) programs. Mexico is offering the vaccine free of charge in 10 of its poorest states, and Nicaragua, a GAVI-eligible country, has introduced the rotavirus vaccine into the EPI system as part of a demonstration project with Merck & Co. Numerous other countries around the world have licensed rotavirus vaccines, allowing them to be sold through the private sector.

To determine efficacy of these vaccines in developing- country settings, clinical trials of both vaccines are planned or are underway in Africa and Asia. If proven efficacious, introducing these vaccines into the public sector EPI programs of low-income countries is a global health priority [4]. The question remains however, is this global prioritization of rotavirus vaccines shared by developing countries where the disease is relatively unknown?

The WHO has issued general guidelines for vaccine introduction which provide countries with a framework for considering the evidence about specific diseases and the operational, technical and policy steps necessary for vaccine introduction [10].

The guidelines indicate that the vaccine introduction process begins by determining the public health priority of a particular disease, suggesting that in countries where the disease is not a priority adoption of the vaccine is not assured. The guidelines recommend conducting qualitative investigations of the public health community and decision makers to gauge the concern about the disease and the priority of the vaccine.

This paper summarizes the results of a formative research survey of public health providers in five low-and middle-income countries to determine levels of knowledge about rotavirus and rotavirus vaccines, perceptions of diarrheal disease as a problem and priority for the public health system, and knowledge of the causes, prevention methods, and treatment of diarrheal disease. The formative research results presented here utilized focus group discussions (FGD) and one-on-one, semi-structured interviews to answer surveys, and were supported by funding provided by the GAVI Alliance in support of the Rotavirus Vaccine Program (RVP). RVP is a partnership between WHO, the U.S. Centers for Disease Control, and PATH, a US-based international health organization. The mission of RVP is to accelerate the availability of rotavirus vaccines in developing countries

The results of the surveys have informed the development of a knowledge translation approach to bridging the gap between a global scientific agenda of vaccine development, and the local public health agenda of diarrheal disease control. We hypothesize that this approach–the Enhanced Diarrheal Disease Control Initiative–may help prioritize diarrheal disease sufficient for consideration of the vaccine evidence and potentially prioritization of the vaccine, as well as consideration of other diarrheal disease interventions, such as zinc treatment, and oral rehydration therapy.

## Methods

These surveys represent formative research. Formative research typically utilizes qualitative methods to extract "information on target audiences beliefs, values, attitudes, knowledge and behaviors related to the health problem of interest, and seeks to answer questions about the context that influences, and is influenced by, these individual factors"[11]. The surveys were conducted in 2006 using focus group discussions and one-on-one interviews in five countries: India, Indonesia, Nicaragua, Thailand, and Ukraine. Participants were assured that their responses would not be attributed to them by name and identities were not recorded. Research methods consisted primarily of open-ended questionnaires designed to develop a general public health perspective of rotavirus and diarrheal disease by pursuing the following formative research objectives:

1. Assess current knowledge and attitudes related to diarrheal disease and its causes, prevention, and treatment, with specific attention paid to rotavirus disease.

2. Explore potential messages and solutions to rotavirus communication challenges.

### Research Subjects

A total of 546 research subjects participated in the study. Table 1 details the number of subjects by country for the focus groups and the one-on-one interviews. Subjects included:

**Table 1:** Number of respondents in interviews and focus group discussions by country

**Country**	**No. of FGD participants (number of FGDs)**	**No. of individual interviews**	**Total respondents**
India (Andhra Pradesh)	75 (17)	25	100
Indonesia	57 (6)	25	82
Nicaragua	136 (15)	50	186
Thailand	44 (8)	41	85
Ukraine	62 (9)	31	93

**TOTAL**	**374 (55)**	**172**	**546**

• Physicians (private and public)

• Regional, district, and community level health workers and their managers

• Pharmacists and drug sellers.

• Nurses, mid-wives, and public health volunteers

• National child health experts, advisors to and officials of the Ministry of Health

• Staff of international organizations involved in child health, such as UNICEF and WHO.

(Table 1)

### Discussion guide

A brief discussion guide was developed to facilitate the FGD and one-on-one interviews (Table 2).

**Table 2:** Discussion Guide

Topic I	Diarrheal disease–knowledge and attitudes
Questions	a. How serious a public health problem is diarrheal disease here?
	b. Relative to other health problems?
	c. What are some of the causes of diarrhea?
	d. What are some ways that diarrheal disease can be prevented?
	e. What are some of the challenges you face in helping parents prevent diarrheal disease?
	f. What are challenges related to treating diarrheal disease?

Topic II	Rotavirus and rotavirus vaccine–knowledge and attitudes

Questions	a. Is rotavirus-related disease a problem here?
	b. How serious a problem is it?
	c. What kinds of health problems do you see which can be attributed to rotavirus?
	d. How can rotavirus disease be prevented?
	e. Have you heard about or seen a vaccine against rotavirus?

## Results

Survey findings revealed information about knowledge, attitudes and practices relating to rotavirus and diarrheal disease. Table 3 provides illustrative and representative comments with explanations below.

(Table 3)

**Table 3:** Representative quotes from focus-groups and interviews

**Quote**	**Respondent**	**Country**
**A. Perceptions and knowledge of diarrheal disease**		

*"The problem of diarrhea is quite serious; it has to do with hygiene habits especially, and with dietary habits."*	MOH Official	Nicaragua
"*It is not an important health problem because of low-mortality rate. Parents would seek early medical consultation. Most cases are mild diarrhea."*	Community Health Nurse	Thailand
"*Lack of knowledge regarding sanitation and hygiene, and myths and beliefs among rural populations about diarrhea leads to seriousness of the cases."*	Public Health Care Provider	India
*"It is a social problem and depends on water supply, and personal nutrition and hygiene."*	Medical School Professor	Ukraine
*"Due to negligence, people do not react immediately to the symptoms of the disease."*	District-level private health care provider	India

**B. Knowledge of rotavirus and rotavirus vaccines**		

*"We don't know what the rotavirus is."*	Nurse	Ukraine
*". . .Maybe a little [knowledge of rotavirus] . . . I only know that it causes diarrhea."*	General practitioner	Thailand
*"Rotavirus is prevailing in western countries only. Diagnosis for rotavirus is not done in India. Not aware of vaccine."*	State level health advisor	India
*"It is a water borne disease. But one does not know about its prevalence in India."*	Public health provider	India
*"Not yet, not yet. I have not been informed about rotavirus diarrhea or the vaccine."*	Policy maker	Indonesia

**C. Other causes of diarrheal disease**		

"*We know that viruses are the main cause of diarrhea, but don't know which*."	Pediatrician	Ukraine
*"The causes of diarrhea are mostly food-borne infections, both bacterial and viruses. Most of our cases are viral."*	General practitioner	Thailand
*"Eating mutton and chicken, eating food without washing hands, and living in unclean surroundings are causes of diarrhea."*	Private Health Care Provider	India

**D. Prevention of Diarrheal Disease**		

*"Invest in campaign programs, especially during the peak season of diarrhea. Collaborate with community health centers focusing on hand washing, clean and safe food."*	Advisor, Ministry of Health	Thailand
*"Dispose dust and garbage on day to day basis. Dispose of infant stools properly and wash hands."*	Village-level Public Health Provider	India
*"In order to prevent diarrhea: exclusive breastfeeding."*	Ministry of Health official	Nicaragua
*"In previous times we had a lot of printing materials and posters regarding prevention of diarrheal disease and personal hygiene, now we don't have these."*	Pharmacist	Ukraine

**E. Diarrheal Disease Treatment**		

*"Oral rehydration therapy is the best."*	Ministry of Health Official	Nicaragua
*"Most physicians don't even realize that decrease in death rates among infants in the past few years is due to broader implementation of rehydration therapy."*	Medical School Professor	Ukraine
*"The challenges [for treatment]: the culture of each individual, self medication, many purge their children and they come in dehydrated, they stop giving them food because they say it hurts them."*	Private physician	Nicaragua
*"Mothers stop breast feeding the child with diarrhea. This leads to seriousness." Public Health care provider, Village level, Indian*	Village Level Public Health Care Provider	India
"*People frequently use self-treatment with antibiotics, and this is bad."*	Pediatrician	Ukraine

**F. Zinc treatment**		

*"Do not know much [about zinc]. It is still under study."*	District-Level Public Health Care Provider	India
*"We have never heard about zinc but think that regular use of yogurts can protect from gastroenteritis."*	Pediatrician	Ukraine

### A. Perceptions and knowledge of diarrheal disease

In all of the countries surveyed it was widely understood that serious diarrheal disease morbidity and mortality is a direct result of dehydration. Differences between the countries emerged in the level of concern about diarrhea and the priority of diarrheal disease as a public health problem.

In India, Indonesia, and Nicaragua, countries with relatively high under-five mortality and high diarrhea-related mortality (Table 4), nearly all participants in interviews and FGD indicated that diarrheal disease was a serious and significant problem in their country, but that as a priority, diarrhea disease control has slipped.

**Table 4:** Under-5 mortality rates and percentage of under-5 mortality due to diarrhea in survey countries

**Country**	**Under-5 Mortality Rate per 1,000 live births (2005)**	**Percentage of under-5 deaths due to diarrheal disease (2000)**
India	74	20
Indonesia	36	18
Nicaragua	37	12
Thailand	21	16
Ukraine	17	1

Source: WHO Statistical Information System, 2007

In these three countries the problem of diarrheal disease was often described as a reflection of larger cultural or socio-economic conditions. Poor hygiene and environmental conditions were seen as the major contributing factors to diarrheal disease, often exacerbated by under-educated or illiterate young mothers. Further understanding of the disease was greatly hindered in all of the countries by a lack of diagnostic capacity to identify the causal pathogens, and a lack of disease surveillance to determine the disease burden.

Despite this concern and the high level of importance placed on diarrheal disease, many expressed a perception that diarrheal disease was no longer receiving the attention it once had, and that previous diarrheal disease control education programs had languished or the focus had shifted to other health issues. In Nicaragua, India, and Indonesia there was a strong interest in renewed emphasis on preventing diarrheal disease through health education.

In Thailand, a middle-income country with lower levels of under-five mortality, diarrheal disease was seen as a relatively insignificant problem due to the wide availability of treatment and a high degree of community awareness of prevention and management methods. This view seems to contradict the data suggesting a relatively high percentage of under-five deaths caused by diarrhea (Table 4). Hospital based physicians in Thailand considered severe cases to be problematic but infrequent. And most community-level health care workers perceived diarrhea as common and manageable, with most respondents at all levels indicating that severe dehydration and death from diarrhea were rare.

In Ukraine, opinions varied significantly as to the seriousness of diarrheal disease as a problem in that country, leading the research team to conclude that there was no consensus about the priority of diarrheal disease in Ukraine. Many of the Ukrainian respondents believed that diarrheal disease was not a major problem, and in instances of outbreaks, was thought to be well managed in in-patient and out-patient settings. However, certain medical and child survival specialists, and public health professionals at the national and regional levels (oblasts), voiced a contrary viewpoint, that diarrhea and dehydration were a serious problem for young children in Ukraine, despite low mortality from diarrhea.

In India, Indonesia, and Nicaragua, while rehydration therapies exist, several respondents indicated that many parents and caregivers wait too long to seek care, or are unable, or unwilling to do so and was an indicator of inadequate education about the disease and its potential severity. Some expressed a view that the delay often stems from parental beliefs that diarrhea is a common aspect of early child development, and was a sign of development and growth, which can lead to delays in care-seeking until severe symptoms occur.

### B. Knowledge of rotavirus and rotavirus vaccines

In all of the countries, except for Nicaragua, awareness about rotavirus was extremely low. In fact, outside of university- or hospital-based pediatric and virology settings, rotavirus was an illness few in the broader public health community had heard of or knew anything about.

Those few with any familiarity with rotavirus did not differentiate it from other causes of diarrheal disease in terms of its transmission, prevention and treatment. They often expressed inaccurate information about the disease, and indicated, incorrectly, that improved hygiene and access to clean water would adequately prevent rotavirus. Many also indicated that ORT was a viable treatment option, however, in the face of severe disease, ORT is very difficult to administer outside of clinical settings due to the profuse vomiting that occurs.

In 2004 and 2005, Nicaragua and several other Central American countries faced an exceptionally severe rotavirus season. Several hundred deaths in the region led to declarations of public health emergencies, and significant public warnings and information campaigns to reduce the number of sick children[12]. This helped sensitize the country to rotavirus, and certainly contributed to increased awareness among providers. Despite this high visibility, a number of participants expressed a lack of awareness about the disease. Not surprisingly, while few had heard of the disease, even fewer were aware of a vaccine or its potential.

Knowledge of rotavirus was so low in all countries that facilitators and interviewers were not able to test potential messages about rotavirus and the vaccine that might be used in building awareness about the disease. The respondents could not effectively respond without further information about the disease and the vaccine.

Regardless of their knowledge of the disease, discussions about rotavirus vaccines revealed some skepticism about whether or not the disease burden sufficiently warranted a vaccine. Even among knowledgeable pediatric specialists, lack of information about disease burden prevented them from making a judgment about the priority of the disease and the vaccine. In addition, in Indonesia and India several participants assumed that the vaccine would be expensive and likely out of reach financially for the poorest populations.

### C. Other causes of diarrheal disease

In almost all instances, discussions about the cause of diarrheal disease centered around the transmission of diarrheal disease, such as poor hygiene and water. Almost no mention was made of specific pathogens, although among physicians and others with advanced public health training, there was acknowledgement that there are viral and bacterial causes.

All of the subjects identified poor sanitation and hygiene behaviors, such as unsanitary food handling and storage, lack of hand washing after latrine use, and improper waste disposal, as the most important and common causes of diarrhea. In addition, lack of access to potable water was also cited as a frequent cause of diarrheal disease.

Respondents in all the countries cited the lack of laboratory diagnostics and surveillance as a barrier to determining the type and causal agent of diarrheal disease, often leading to potentially inappropriate treatment recommendations.

### D. Prevention of diarrheal disease

Prevention was closely linked to cause. Public education was cited as the most important prevention effort, with particular focus on parents, in order to improve hygiene practices and convey the importance of clean water. In addition, respondents cited education about exclusive breastfeeding of infants as another important preventive approach.

In Ukraine, educating food handlers in restaurants and shops was thought to be the most effective and efficient intervention to prevent diarrheal disease. In Nicaragua, there was interest in behavior change efforts aimed at parents to improve overall hygiene. In India, and to some extent Indonesia, there was strong interest within the public health community in renewing the focus on diarrheal disease prevention at the community and national levels.

In all of the countries findings indicated that community education campaigns were no longer being conducted to the extent and with the frequency they once were.

### E. Treatment

The use of oral rehydration solution was frequently cited as a successful treatment. In India, several respondents indicated that despite past public education efforts, use of oral rehydration therapy was relatively low. In Nicaragua, some participants indicated that many parents did not use oral rehydration solution appropriately.

In India, Thailand, and Indonesia, the use of various oral rehydration therapies such as milks, tea, water, juice and other liquids were often mentioned as important treatment methods.

In several countries ineffective and potentially dangerous diarrheal disease management practices were said to be practiced. In Nicaragua, India and Indonesia, in particular, purging with laxatives and other agents, stopping breastfeeding, massage, and withholding food and water were said to be common practices, particularly among low-literate populations. In all of the countries, inappropriate use of antibiotics and anti-diarrheal medications were also frequently mentioned.

### F. Zinc treatment

Recent studies indicate that treating diarrhea with zinc can significantly reduce the burden of dehydration caused by diarrheal disease among children under 5 in developing countries [13]. These studies suggest that 20 mg of zinc per day over 10–14 days at the onset of diarrhea can significantly reduce the duration and severity of the illness, stool output, and the need for hospitalization. Zinc may also prevent future episodes of the disease for up to 3 months, and has been shown to reduce the inappropriate use of antibiotics.

Questions and considerations of zinc treatment were added late to the discussion guide, as a result, discussions about zinc were conducted only in the interviews and FGD held in India, Ukraine, and Thailand. Participants were asked about their knowledge of zinc as an intervention for controlling diarrhea. Most indicated little or no knowledge of zinc as a treatment for diarrhea.

## Discussion

These formative survey results confirm what has been reported elsewhere, that rotavirus is a relatively unknown disease [14], and in developing countries, except among a narrow group of highly specialized medical professionals, few in the public health community have heard of or are knowledgeable of rotavirus. This lack of awareness by the public health community is a significant barrier to the prioritization of the disease, which WHO states is the first step towards consideration of the evidence for new vaccines [10].

Building awareness of rotavirus in the public health communities of developing countries to a level sufficient for consideration of the evidence and subsequent prioritization of the disease is a challenging task. Passive measures to present rotavirus-specific evidence through peer-reviewed articles in the scientific literature and presentations, usually in academic or scientific settings, are necessary but likely insufficient for prioritization. A search of rotavirus on the National Library of Medicine's PubMed database identifies over 7,000 peer-reviewed articles have been published since rotavirus was discovered in 1973, and undoubtedly countless presentations made, yet the disease remains virtually unknown within the public health community of developing countries where the impact of the disease is most severe. Clearly a gap exists between the global evidence base about rotavirus and local knowledge and awareness.

The challenge of bridging this gap is compounded by a significant slowdown in the broader diarrheal disease control effort at the global and national levels. While the problem of diarrheal disease remains a significant cause of morbidity and mortality, the surveys indicate a widespread belief that diarrheal disease programming has diminished in recent years. This perspective is supported by evidence suggesting that coverage of existing diarrheal disease control interventions such as oral rehydration therapy has leveled off, or in some countries, actually decreased [15]. One reason for this may be that in many countries the Control Diarrheal Disease programs, which focused resources and attention on preventing and treating diarrheal disease so successfully in the 1980's and 1990's, have been subsumed by the Integrated Management of Childhood Illness (IMCI) approach. This may have had the unintended consequence of attenuating diarrheal disease control in favor of a more generalized child health effort, where a range of child health messages and resources are co-mingled.

Secondly, the global attention focused on AIDS, TB and malaria, as evidenced by the significant global and national resources being directed to those concerns, has had the effect of marginalizing other child health issues, including diarrheal disease, and progress towards improved child survival in developing countries has slowed [16].

### Renewing commitment to diarrheal disease control

What is needed is a renewed commitment to reducing the burden of diarrheal disease among children in poor countries, and the development of rotavirus vaccines and zinc treatment provide a catalyst for this renewal which in turn may accelerate their introduction.

We hypothesize that a "knowledge translation" approach could provide a catalytic framework for rebuilding momentum for diarrheal disease control and overcoming the lack of awareness of rotavirus and zinc by demonstrating the relevance of these new interventions to the broader public health agenda of diarrheal disease control.

Knowledge translation is a nascent global health paradigm with several definitions, but can be summarized as a theoretical process of bridging global scientific evidence with local public health experience, practice and policies to move evidence into policy and action [17,18]. This bridging relies partly on "contextualizing" the evidence, which a panel of knowledge translation experts convened by the WHO suggested, "implies that evidence is plural and that the implementability of good 'global' evidence must be triangulated with local knowledge" [17].

The results of this survey indicate that, while rotavirus is little known and as a result not considered a major priority, diarrheal disease remains a significant concern in poor countries, as represented by Nicaragua, Indonesia and India. The results of the survey also indicate a broad understanding of the causes, prevention, and treatments of diarrheal disease generally. Dirty water, poor sanitation and hygiene practices were frequently implicated as the cause of diarrhea. While these are not necessarily the causes of diarrhea, they are certainly the transmission routs of pathogens, and this study indicates that past efforts to educate the public health community about the importance of improved water and sanitation have been successful. Similarly, oral rehydration therapy, home management, and breastfeeding, were frequently mentioned as critical components to controlling diarrhea.

The strong knowledge base of existing diarrheal disease control strategies provides the appropriate contextual framework for reaching out with information and evidence about rotavirus and rotavirus vaccines, as well as other diarrheal disease control interventions, as opposed to launching independent, vertical approaches to knowledge building and demand creation. By integrating information about rotavirus with other diarrheal disease interventions, it may be possible to create a corresponding priority-by-association for rotavirus. Moreover, the discussions revealed that in the poorest countries there is a desire for renewed attention to diarrheal disease, further indicating receptiveness for viable solutions to diarrheal disease.

### Support for an integrated approach

The notion of integrating rotavirus into the broader landscape of diarrheal disease to build greater association and awareness is supported by the findings of the Diseases of the Most Impoverished (DOMI) Program of the International Vaccine Institute. The DOMI Program was established to accelerate the development and use of existing and future enteric vaccines against typhoid fever, cholera, and shigellosis. Through extensive surveys in developing countries, DOMI researchers found that county-level decision makers consider enteric vaccines, of which rotavirus is one, as only one component of diarrheal disease control efforts that include everything from public education to improvements in water, sanitation and hygiene, as well as vaccines [19].

An integrated approach also provides opportunities to reinforce existing interventions, such as ORT, which as mentioned above, suffers from stagnating coverage, but because of its effectiveness should remain a cornerstone of any diarrheal disease control effort. This approach may also help overcome the "replacement effect" in which some caregivers may assume incorrectly that one intervention, such as a rotavirus vaccine, replaces the need for other interventions. This phenomenon was of concern initially to researchers conducting field trials of zinc treatment, who believed that caregivers might replace the use of ORS with zinc, when in fact both interventions are necessary. To overcome this, they co-promoted zinc and ORS, and found that this integrated approach resulted in an increased use of both interventions [20].

Rotavirus vaccine will only prevent rotavirus, it is not intended to prevent other forms of diarrhea, and as a result co-promotion or an integrated approach emphasizing the continued use of other interventions for preventing and treating other forms of diarrhea is warranted. In fact, WHO's Strategic Advisory Group of Experts has recommended the development of strategies to prevent any misinterpretation that rotavirus vaccine could prevent other childhood diarrhea [21]. Integrating rotavirus vaccines into a broader effort could help minimize the risk of replacement.

Instituting an integrated approach also finds support in the Global Immunization Vision and Strategy (GIVS), the WHO and UNICEF's 10-year strategic plan for expanding access to existing vaccine and introducing new vaccines. GIVS identifies as one of its four strategic areas the integration of immunizations with other health interventions [22]. Clearly, including rotavirus vaccine in a broader context of diarrheal disease interventions is supportive of this strategy.

## Recommendations

### Towards an integrated diarrheal disease control approach

**Figure 1:**
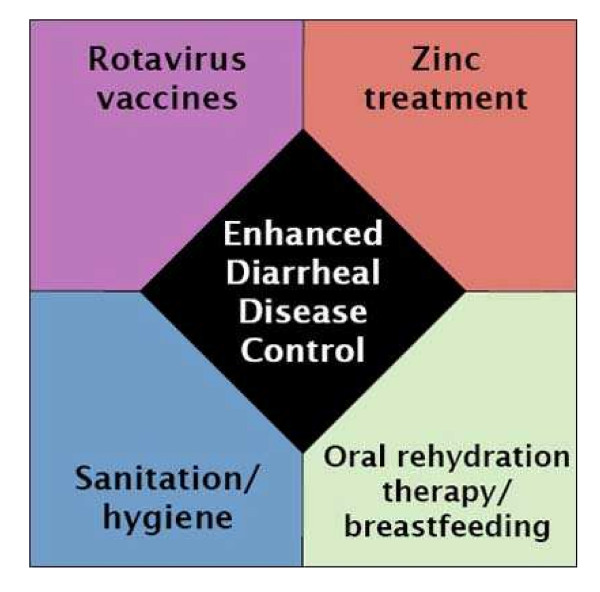
Enhanced Diarrheal Disease Control Framework.

Based on the results of this survey, the authors have proposed a knowledge translation strategy that raises awareness about new and existing diarrheal disease control measures using an integrated framework. This approach–the Enhanced Diarrheal Disease Control Initiative (EDD)–integrates information and evidence about rotavirus and rotavirus vaccines, zinc treatment, and the new formulation of low-osmolarity ORS, as well as information to reinforce the use of existing interventions such as ORT, exclusive breastfeeding, and improvements in sanitation and hygiene (Figure 1). This framework is also designed to accommodate future interventions such as vaccines for cholera, typhoid, ETEC, and *Shigella*, or clean water technologies.

The goal of EDD is to stimulate decision makers in developing countries to consider evidence about new and existing interventions for diarrheal disease control. It relies on the establishment of a public health coalition representing a range of disciplines concerned with diarrheal disease (pediatrics, nutrition, immunization, sanitation, etc.) to begin the knowledge translation process aimed at the following objectives:

1. Emphasize to decision-makers the importance of diarrheal disease as a public health priority and the need for new interventions.

2. Present the evidence about new interventions for diarrheal disease control to appropriate stakeholders in a manner reflective of local knowledge, experience, and practice.

3. Reinforce the importance of existing diarrheal disease interventions, such as ORT, breast feeding, and improved sanitation and hygiene

With support from the GAVI Alliance, EDD is being piloted in several countries in Latin America and Asia. Early indications suggest that local diarrheal disease coalitions have used the concept successfully to raise awareness for the new interventions throughout the public health system, stimulate policy changes such as the listing of zinc as an essential drug, and in one country, may have spurred the decision to undertake a demonstration project of rotavirus vaccine. Perhaps most importantly, diarrheal disease is once again near the top of the public health agenda in these countries, EDD having catalyzed consideration of both the problem and the solutions. Formal impact evaluations of EDD pilot projects are planned. We believe that in some countries the EDD strategy will help RVP achieve its mission of accelerating introduction of rotavirus vaccine by raising the awareness of the vaccines in the context of a renewed diarrheal disease control strategy.

## Limitations

This qualitative study was conducted to develop a generalized public health view of rotavirus and diarrheal disease in developing countries, particularly with regard to the priority and concern for diarrheal disease and its causes. As a result, we did not stratify nor quantify the data, choosing instead to find common themes and perspectives.

As with many qualitative studies, the responses are open to bias from those collecting the information. In addition, FGD are often limited by the potential of participants to be unwilling to say certain things in public.

Greater attention should be paid in future studies to quantifying levels of knowledge and attitudes by particular sectors of the public health community in order to further refine communication, education and information programs.

Finally, the paper recommends a knowledge translation strategy, fully accepting the fact that there are few if any studies indicating that knowledge translation is effective in accelerating adoption of policies or interventions. However, the authors believe that as a theory knowledge translation has merit. The Enhanced Diarrheal Disease Control Initiative adopts the knowledge translation theory to achieve the desired objectives of rebuilding momentum for diarrheal disease control, accelerating introduction of new interventions, and reinforcing use of established interventions.

## Conclusion

RVP was established to accelerate the introduction of rotavirus vaccines but found that the lack of knowledge of rotavirus by the public health community in some developing countries was insufficient to prioritize the vaccine as needed for uptake. In addition, it was found that the broader diarrheal disease control effort has lost considerable momentum, and is currently a weak platform for advancing new interventions.

The availability of research evidence that includes rotavirus disease burden estimates and clinical trial results may be enough for *global *prioritization, even if the disease are relatively obscure or unknown. But in developing countries where lack of familiarity with rotavirus and pre-existing priorities compete for scarce resources, global evidence alone may not be enough to guarantee prioritization of the disease and uptake of the vaccine.

Knowledge translation offers one potential approach to link global evidence with a local context of diarrheal disease control knowledge, experience and practice in order to build an association between rotavirus and the expressed priority of diarrheal disease.

The EDD approach is designed to rekindle interest in diarrheal disease control at the country level, and to put knowledge translation into practice by stimulating the prioritization and uptake of rotavirus vaccines, as well as zinc treatment, and to reinforce use of existing diarrheal disease control measures and prevent child deaths.

## Competing interests

The author(s) declare that they have no competing interests.

## Authors' contributions

ES developed the survey instrument, assessed the final data from each country, and was lead author. SW assisted with developing the survey instrument, site selection, and managed data collection from Thailand and India. JB managed data collection, analysis, and reporting for research in Nicaragua. KG managed data collection, analysis, reporting, and translation from research in Ukraine. LC managed research activities in Indonesia and editing of Indonesia reports. JW assisted with data analysis from Nicaragua and translation. All authors have read and approved this manuscript.

## Pre-publication history

The pre-publication history for this paper can be accessed here:



## References

[B1] Kosek M, Bern C, Guerrant RL (2003). The global burden of diarrhoeal disease, as estimated from studies published between 1992 and 2000. Bull World Health Organ.

[B2] Parashar UD, Gibson CJ, Bresse JS, Glass RI (2006). Rotavirus and severe childhood diarrhea. Emerg Infect Dis.

[B3] Velazquez FR, Matson DO, Calva JJ, Guerrero L, Morrow AL, Carter-Campbell S, Glass RI, Estes MK, Pickering LK, Ruiz-Palacios GM (1996). Rotavirus infections in infants as protection against subsequent infections. N Engl J Med.

[B4] Organization WH (2003). Rotavirus vaccines, an update. Weekly Epidemiologic Record.

[B5] GAVI Alliance (2003). GAVI and the Vaccine Fund announce $60 million boost to accelerate development of lifesaving vaccine.. http://www.gavialliance.org/media_centre/press_releases/2003_02_11_en_press_110203.php.

[B6] Vesikari T, Matson DO, Dennehy P, Van Damme P, Santosham M, Rodriguez Z, Dallas MJ, Heyse JF, Goveia MG, Black SB, Shinefield HR, Christie CD, Ylitalo S, Itzler RF, Coia ML, Onorato MT, Adeyi BA, Marshall GS, Gothefors L, Campens D, Karvonen A, Watt JP, O'Brien KL, DiNubile MJ, Clark HF, Boslego JW, Offit PA, Heaton PM (2006). Safety and efficacy of a pentavalent human-bovine (WC3) reassortant rotavirus vaccine. N Engl J Med.

[B7] Ruiz-Palacios GM, Perez-Schael I, Velazquez FR, Abate H, Breuer T, Clemens SC, Cheuvart B, Espinoza F, Gillard P, Innis BL, Cervantes Y, Linhares AC, Lopez P, Macias-Parra M, Ortega-Barria E, Richardson V, Rivera-Medina DM, Rivera L, Salinas B, Pavia-Ruz N, Salmeron J, Ruttimann R, Tinoco JC, Rubio P, Nunez E, Guerrero ML, Yarzabal JP, Damaso S, Tornieporth N, Saez-Llorens X, Vergara RF, Vesikari T, Bouckenooghe A, Clemens R, De Vos B, O'Ryan M (2006). Safety and efficacy of an attenuated vaccine against severe rotavirus gastroenteritis. N Engl J Med.

[B8] Organization WH (2006). Conclusions and recommendations from the Strategic Advisory Group of Experts to the Department of Immunizatoin, Vaccines and Biologicals. Weekly Epidemiologic Record.

[B9] Alliance GAVI (2007). Rotavirus Vaccine. GAVI Alliance.

[B10] Organization WH (2005). Vaccine Introduction Guidelines. http://www.who.int/vaccines-documents/DocsPDF05/777_screen.pdf.

[B11] Newes G, Helitzer DL, Caulfield LE, Brown KH (2000). Theory and practice:  applying the ecological model to formative research for a WIC training program in New York State. Health Education Research.

[B12] ProMED-Mail (2005). Viral Gastroenteritis Update 2005. http://www.promedmail.org.

[B13] Bhutta ZA, Black RE, Brown KH, Gardner JM, Gore S, Hidayat A, Khatun F, Martorell R, Ninh NX, Penny ME, Rosado JL, Roy SK, Ruel M, Sazawal S, Shankar A (1999). Prevention of diarrhea and pneumonia by zinc supplementation in children in developing countries: pooled analysis of randomized controlled trials. Zinc Investigators' Collaborative Group. J Pediatr.

[B14] Glass RI, Parashar UD, Bresee JS, J G, Turcios R JB, CA DQ (2004). Rotavirus Vaccines. Vaccines:  Preventing Disease and Protecting Health.

[B15] Forsberg BC, Petzold MG, Tomson G, P. A (2007). Diarrhoea case management in low- and middle-income countries--an unfinished agenda. Bull World Health Organ.

[B16] Editorial (2003). The world's forgotton children. Lancet.

[B17] Organization WH, The Canadian Coalition for Global Health Research Canadian International Development Agency German Agency for Technical Cooperation (GTZ) (2005). Bridging the "Know-Do" Gap:  Meeting on Knowledge Translation in Global Health: 2005/10/10..

[B18] Health CI (2004). The CIHR Knowledge Translation Strategy 2004-2009:  Innovation in Action. http://www.cihr-irsc.gc.ca/e/26574.html.

[B19] DeRoeck D, Clemens JD, Nyamete A, Mahoney RT (2005). Policymakers' views regarding the introduction of new-generation vaccines against typhoid fever, shigellosis and cholera in Asia. Vaccine.

[B20] Gilroy K, Kuszmerski NWP (2005). Lessons learned in a pilot introduction of zinc treatment for childhood diarrhea in Bougouni District, Mali.

[B21] Organization WH, Fund UNC (2005). Global Immunization Vision and Strategy, 2006-2015.

